# Cost-Benefit Analysis of an Intervention in Divorced Parents: Implications for Society, Public Administrations and Family Visitation Centers

**DOI:** 10.3390/ijerph19063484

**Published:** 2022-03-15

**Authors:** Leire Alcaniz, Ana Martínez-Pampliega, Marta Herrero

**Affiliations:** 1 Department of Finance and Economics, Deusto Business School, University of Deusto, 48007 Bilbao, Spain; 2 Department of Psychology, Faculty of Health Science, University of Deusto, 48007 Bilbao, Spain; martinez.pampliega@deusto.es (A.M.-P.); m.herrero@deusto.es (M.H.)

**Keywords:** cost-benefit, divorce, mental health, efficiency, children, mental health outcomes, divorced parents, program evaluation, quasi-experimental

## Abstract

Families going through conflictive divorce processes are at increased risk of developing mental health problems. The Egokitzen program is a group intervention for parents who have undergone a divorce process, funded by the public administration. Budgetary constraints cause funding institutions to be interested in the effectiveness and economic efficiency of these programs. Therefore, the objective of this research is to carry out an efficiency analysis of the Egokitzen program, implemented by family visitation centers in Spain, through a cost-benefit analysis, to determine whether the positive impact on symptomatology (measured using CBCL and SCL-90 instruments) is translated into a positive economic impact for society. A sample of 382 parents participated. Costs will be first identified and valued; secondly, benefits achieved with the program will be identified through a prevalence analysis and, finally, the cost-benefit comparative analysis will be carried out. Additionally, a sensitivity analysis will be performed. The results obtained in the analysis indicate that for every euro spent on this program, the public administration and society save 3.10 euros in future interventions through medical costs and productivity losses. The study has practical implications for public administration, organizations, and the family visitation centers that implement the program.

## 1. Introduction

The United Nations has focused on the 17 most important global problems through the Sustainable Development Goals (SDG). Specifically, promoting good health and well-being is one of these goals (SDG3). Given that mental health problems affect 25% of the world’s population and that problems such as anxiety or depression are one of the leading causes of disability in many countries, mental health care is one of the major challenges facing European health systems [1]. In fact, the situation due to COVID-19 has changed people’s lifestyles, causing the number of people with mental health problems to increase or the symptoms in people who already had them to intensify [2,3].

In this sense, families, both parents and children, who go through conflictive divorce proceedings are a population at risk for the development of mental health problems. Studies show that divorces create psychological problems, through externalizing and internalizing manifestations, both in parents and children [4,5,6,7].

This reality is significantly prevalent, given that the number of divorces at the European level amounts to almost one million families per year. In Spain, this figure reached 95,254 divorces during 2018, declining to 77,200 in 2020 (due to the COVID-19 confinement and suspension of procedural deadlines). Of the total of divorced couples, more than 60% at the European level and 45% at the Spanish level had at least one child [8,9]. Given these figures and the effect of the current pandemic situation on families, an important part of the population has or will have to face a divorce process [10]. It is therefore necessary to create programs to reduce the psychological consequences of these processes.

Studies at the international level have obtained results confirming that conflictive divorce is a fundamental stressor in children, with the potential to cause long-term health problems [11]. The destructive interparental conflict that accompanies these processes also indirectly affects relationships with children and is a key factor to understand their psychological health [12,13]. That is why, since the mid-1990s, many of the programs created have been aimed at reducing child symptomatology, the most frequent being those that work directly with the parents so they can help their children cope with divorce adequately or support them during this transition. These programs include Children in the Middle [14] and New Beginnings [15], among others. The challenge is currently to study the efficacy and effectiveness of these programs. While these studies focus on the American setting, the Egokitzen program, the subject of this research, is one of the first to be developed in Europe, specifically in Spain.

The Egokitzen program is a group intervention for parents who have undergone a divorce process. The nature of the program is preventive, psycho-educative, and with a systemic orientation of family functioning. The program achieved good results in a university laboratory context [16] and the community context [17], showing a reduction of parental symptomatology and, indirectly, of child symptomatology. However, besides being effective in terms of mental health, public agencies are also interested in whether the programs in which they invest public money are economically efficient [18]. In fact, many of these mental health programs are funded by the public administration. Given the budgetary constraints that are normally considered in the public sector, the financing of these programs depends increasingly on a positive outcome in the cost-benefit analysis [19].

Therefore, the objective of this research is to carry out an efficiency analysis of the Egokitzen program through a cost-benefit analysis, to determine whether the positive impact on internalizing and externalizing symptomatology has a positive economic impact on society.

## 2. Materials and Methods

### 2.1. The Intervention—The Egokitzen Program

The Egokitzen program intervention is performed in a group format. Only one of the members of the divorced couple participates in the group. Each parent group participates in 11 weekly one-and-a-half-hour sessions, with a dynamic methodology (group activities, role-playing, etc.). These sessions focused on three areas: divorce and its impact, interparental conflict, and parenting. Before the initiation, the facilitators who are in charge of the parent groups receive a 5-h instruction and the necessary documentation to work with the groups.

### 2.2. Presentation of the Study

The study was carried out with the collaboration of 13 family visitation centers (FVCs) that are located in 10 of the 19 Spanish autonomous communities. FVCs were part of the National Federation of Family Visitation Centers (FEDEPE-Federación Nacional de Puntos de Encuentro) which brought together 33 centers at national level. Up to 13 centers chose to participate in the research. The study focused on families with children with high interparental conflict, who attended the FVC by judicial referral. Interparental conflict was measured considering hostility, detachment and escalating distress through the judicial referral report and interviews conducted by the professionals of the FVCs (case history). The inclusion criteria for the study were as follows: the parents were legally separated, did not live in the same household, did not present an active restraining order for gender-based violence, and did not have severe pathological disorders. The FVCs obtained permission from the relevant public administrations to carry out the program. The FVC staff invited the users who met these conditions to participate in the Egokitzen program. The interventions of the study took place between 2016 and 2019.

The research used a quasi-experimental study. Participants were divided into the intervention group (IG) and the wait-list comparison group (CG). Descriptive information was collected from the parents by the FVC staff and data of the main variables of the study through the tests completed by the parents. Both groups of parents (IG and CG) completed the pre- and post-tests, and the IG group completed the questionnaires after 6 months and 12 months.

We used the following instruments to measure the symptomatology of parents and children.

Symptoms Checklist (SCL-90 [20] in the Spanish version of González de Rivera et al. [21]) to analyze the dimensions of Interpersonal Sensitivity, Depression, and Anxiety in the parents. Each dimension consists of 9, 13, and 10 items, respectively, which are presented on a 5-point Likert scale ranging from 0 (not at all) to 4 (a lot). The Cronbach alpha was α = 0.96.

The Child Behavior Checklist (CBCL [22]) was applied to analyze the dimensions of Somatization, Anxiety-Depression, and Aggressiveness in children, as informed by the parents. It consists of 12, 13, and 18 items, respectively, and dimensions are rated on a 3-point Likert scale ranging from 0 (not true), 1 (sometimes true) to 2 (very often or quite often true). Cronbach’s alpha was α = 0.92.

The final sample of the study included 197 people. Initially, the FVCs contacted 1538 users who met the above-mentioned conditions to participate in the study. After explaining the program, 450 people (29.3%) showed an interest in participating, of whom 382 (84.5%) attended a personal interview.

**Figure 1:**
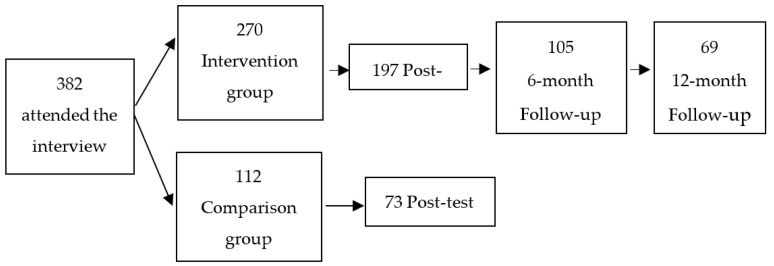
Participant flow chart.

From the initial sample of 382 participants who, after the interview, signed informed consent and completed the evaluation protocol, 112 were part of the CG, and 270 participated in the IG. Of the 112 control participants, 73 people (attrition = 35%) completed the post-treatment questionnaire. Of the 270 intervention participants, 197 (attrition = 24%) responded to the test after the intervention. Of the 197 people, 105 completed the questionnaire at six months and 69 also completed the questionnaire 12 months after the intervention (see Figure 1). Some of the reasons to arrive to the final sample were practical reasons, such as work schedule, group schedules or children’s difficulties. The number of drop-outs between pretest, posttest, 6-month and 12-month follow-ups was due to the length of the evaluation protocol or the lack of motivation to complete it as there was no economic or other type of compensation. There were no differences between the groups (those who dropped out or who did not) in any of the variables considered.

Of the parents who participated in the study, 61% were mothers (see Table 1). The time since divorce was more than 3 years in 48% of the cases, 2 to 3 years in 13%, 1 to 2 years in 20% of the cases, and less than one year in 18% of the cases. Participants had an average of 1.5 children, and 22% had joint custody. For the purposes of the study, only one adult and one child per family were included. The average age of parents was 41.18 (SD = 6.49) and the average age of children was 8.40 (SD = 4.30).

The study was approved by the Ethics Committee of the University of Deusto (ETK-7/16-17). Participants were informed of the study from the outset and signed the consent document. None of the people involved in the intervention received an incentive to participate in the study.

### 2.3. Cost-Benefit Analysis

**Figure 2:**
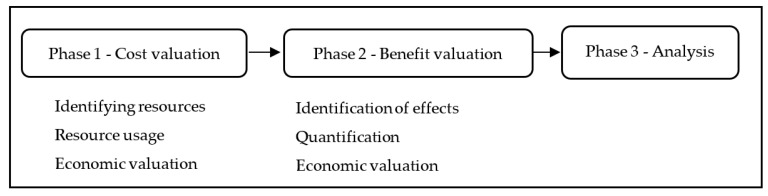
Phases of the cost-benefit analysis.

The cost-benefit analysis was carried out in three consecutive phases (see Figure 2). In the first phase, we assessed the costs of implementing the program, following the methodology recommended by Foster et al. [19]. Following this method, first, the different types of resources that were required were identified. In the second phase, the usage of those resources was measured. And finally, the usage of resources was valued in monetary units. This program presents two stages, one to train the trainers who will carry out the intervention and a second stage of the implementation of the intervention.

The resources required to carry out the training of the FVC workers are the trainers who taught the training course, trainers’ transportation, course materials, and course participants (FVC workers), considering this as an opportunity cost because while receiving the formation, they were not carrying out other normal activities of their position. The resources required to carry out the interventions are: FVC professionals, materials (reflection documents, theoretical reading content, cases with different situations to discuss in class, the evaluation protocol, pens or photocopies), and administration services for program management. Table 2 presents the resources, the variables used to measure them, and the monetizing measures used, at both stages.

In the second phase, the effects achieved through the implementation of the Egokitzen program were valued. First, the main benefits of the program were identified, and then, quantified and, thirdly, valued in monetary units. In the case of the Egokitzen program, the identified benefits were a statistically significant improvement in the parents’ depressive symptomatology and the children’s anxiety when the parents’ global symptomatology improved, as can be seen in Martínez-Pampliega et al. [17]. Situations not statistically significant were not considered in this phase.

The effects were then quantified. Based on the parents’ depressive symptomatology, a prevalence analysis was performed to observe how many IG parents’ depressive symptomatology improved or worsened compared to the control group. Based on the Spanish population standards of the SCL-90 [21], we established cut-off points such that people whose personal score (*p*) exceeded the standardized score (T) of 18.2 in mothers and 14.04 in the fathers were considered to have high levels of depression. This coincides with the 85th centile of those standards. Concerning child anxiety, we observed how many IG parents’ global symptomatology improved. Based on weighted data from the global symptomatology subscales included [21], we established cut-off points such that people whose personal score exceeded the standardized score (mean plus one standard deviation) of 31.04 in mothers and 23.68 in fathers were considered to have high levels of global symptomatology. Next, a prevalence analysis was performed to determine how many children improved or worsened their anxiety when their parents had improved their global symptomatology, comparing the pre-test and post-test results.

Third, the effects were valued in monetary units. Several studies have calculated the costs of depression in different countries, through the savings both of direct (primary care, specialist, pharmacological) and indirect (loss of productivity) costs produced by a reduction in symptomatology [23,24]. In this case, we used the cost savings calculated by Salvador-Carulla et al. [25], who carried out a study in a Spanish region with a health system similar to that of the regions that participated in this research. Concerning children’s anxiety, we used the cost savings calculated by Gustavsson et al. [26]. Their study of direct and indirect costs was carried out for 30 European countries, including Spain. Direct costs include components such as visits to a general practitioner, hospital stays in mental health units (general hospital) and in acute care units (psychiatric hospital), visits to psychiatrists and psychologists, nurse and social work visits, or pharmacological treatment (dispensed prescriptions of anti-depressants, antipsychotics, anxiolytics and antiepileptic). While indirect costs include items such as, temporary disability for depression diagnosis in terms of working days lost and permanent disability for depression (early retirement). Although there are more recent cost-of-illness studies, such as Pella et al. [27], the studies conducted by Salvador-Carulla et al. [25] and Gustavsson et al. [26] are closer to the cost structure of the Spanish health institutional framework and, therefore, this work follows them. All figures were converted to monetary units at the time of this study to be comparable, using variations in the Consumer Price Index (CPI) [28].

The final impact of the program was determined in the third phase taking into account the benefits achieved and the costs required. This shows whether the funds invested in the Egokitzen program had a positive economic impact on society.

Finally, a sensitivity analysis was carried out to analyze the effects of the global outcome of the project, changes both in the costs and in the estimation of benefits.

## 3. Results

### 3.1. Cost Analysis

First, the costs were calculated. Table 3 lists the costs of the program by stage (training or intervention). The ‘Total cost (€)’ column corresponds to the total number of FVCs. Sometimes, the training groups were made up of professionals from more than one center. As shown in Table 3, the total costs amounted to 57,236.42 euros, with 11,099.81 euros for training costs and 46,136.61 euros for the cost of the intervention with the parents. Within the intervention costs, the main cost is that of the FVC professionals, which accounted for 96.6% of the total. This makes sense, because the intervention proposed in the Egokitzen program is based on human capital, which is made up of a team of FVC professionals, and not on medical tests or medicines, as can occur in other types of interventions.

### 3.2. Benefits

To assess the benefits of the program economically, first, a prevalence analysis was performed. The previous study [17] indicated that there was a significant improvement in the parents’ depressive symptomatology and the children’s anxiety when the parents’ global symptomatology improved. Once this is demonstrated, a prudent principle was followed when assessing the positive impact on the participants. For this purpose, we focused on those parents whose responses to the test (*p*) had a score higher than the 85th centile (T) (18.2 in the mothers and 14.04 in the fathers), indicating very high depressive symptomatology, to determine whether they improved after the intervention, and on those parents where the opposite occurred and they worsened after the intervention (*p* < T at pre-test and *p* > T at post-test). The prevalence analysis in Table 4 shows how many IG parents improved or worsened their depressive symptomatology compared to the control group. The IG was compared with the CG to incorporate the effect of spontaneous recoveries or worsening. Concerning risk, through the intervention, 11.74% of the people were no longer considered to be at risk (they improved), and 2.39% went from not being at risk to being at risk (they worsened). Therefore, we can infer that the net effect was that 9.35% of people participating in the program improved. 

As discussed in the methodology section, the study of Salvador-Carulla et al. [25] was used to quantify the cost of depression. The cost amounted to EUR 1800 per year in 2006 (or 2206.8 in euros at the time of the intervention), in which both direct and indirect costs are considered. Previous studies show that depressive symptoms tend to persist over time. According to Layard et al. [29], it is reasonable for the positive effects—in this case, the alleviation of depressive symptomatology—to last for at least 2 years.

Concerning improvements in children’s anxiety when the parents’ overall symptomatology improved, first, we determined which parents could be considered to be suffering from a very high overall symptomatology (higher than the mean plus one standard deviation, which represents a score of 31.04 in the mothers and 23.68 in the fathers) at pre-test and a score below that at post-test; that is, their symptomatology improved. Once the families where parents’ improvement were identified, we proceeded to study how many children improved their anxiety symptoms between pre-test and post-test, and how many worsened. Table 5 shows how many children improved or worsened in anxiety symptomatology compared with the control group, to incorporate the effect of spontaneous recoveries or worsening. Of them, 8.06% improved thanks to the intervention and −24.19% worsened, that is the intervention group worsened less than the control group. It follows, therefore, that the net effect is that 32.25% of people involved in the Egokitzen program improved or at least decreased the likelihood of getting worse compared to not participating in the program.

As explained in the methodology section, data from the study of Ander Gustavsson et al. [26] were used to assess monetarily the benefit of the program concerning children’s anxiety. The cost of annual anxiety per person amounted to 997 euros in 2010 (or 1108.66 euros at the time of the intervention), in which both direct and indirect costs were distinguished. In this case, considering they are children, we only considered the direct healthcare costs and direct non-medical costs. We did not take into account indirect costs that refer to lost productivity, as children do not work. According to Gustavsson et al. [26], these direct costs generally account for 62.40% of the total costs. Therefore, the cost of anxiety in children would amount to 691.76 euros per person per year. Several studies show that interventions on anxiety in children last approximately half a year [30,31], so only costs of half a year were considered.

### 3.3. Cost-Benefit Analysis

Table 6 shows the final results of the program. As can be seen, such an intervention can be a net profit for society of 35,510 euros per 100 people. That is, for every euro spent on this program, society recovers 3.10 euros. Furthermore, taking into account the costs of training the professionals of the FVCs, the result is still positive (2.50 euros of benefit per euro spent).

### 3.4. Sensitivity Analysis

When performing the sensitivity analysis, the assumptions used in the study were modified to make them more demanding both for the costs and benefits.

As noted in the costs section, the cost with the greatest impact is that of human capital. For the study, in the cost of staff per hour, it was considered that the intervention was carried out by FVC professionals within their working day. In the absence of FVCs, this kind of service should be provided by private cabinets of psychology, whose cost is higher than the one in the FVCs. In this case, the cost of personnel would be twice as much. Even so, the result would still be positive. For each euro invested, 1.66 would be recovered (see Table 7).

On the side of the benefits, assuming that the negative effects of depressive symptomatology lasted only one year instead of two, and those of anxiety 3 months instead of half a year (although the data collected show that there was an improvement in participants’ symptomatology at 6 and 12 months), the result would still be positive. For each euro invested, 1.55 would be recovered (see Table 7).

Table 8 shows a summary of the results obtained with the different assumptions made during this research.

## 4. Discussion

The objective of this study is to carry out an efficiency analysis of the Egokitzen program through a cost-benefit analysis, to determine whether the positive impact on the internalizing and externalizing symptomatology generated by the program leads to a positive economic impact on society. The results obtained show that the program is not only effective, as it achieves a positive impact on the symptomatology of both parents and children, but is also efficient. The economic valuation made of the profits obtained exceeds the costs of carrying out the intervention, obtaining a return of EUR 3.10 for each euro invested in the program. According to the analysis carried out, this means that by investing 1 euro in the Egokitzen program, the public administration and society save 3.10 euros in future interventions through medical costs and productivity losses.

The study has clear practical implications. It is shown that this program is not only beneficial in terms of improvement of participants’ symptomatology but also in economic terms, as it represents net cost savings. This conclusion is relevant to the public administration working with budgetary constraints, specifically for those managers who decide which programs should be supported. Cost-benefit analysis can help them in this decision-making.

It also has implications for businesses and society, as, on the one hand, the symptomatology of citizens improves, thereby reducing sick leave, improving worker productivity, and contributing positively to the competitiveness of companies and organizations.

Third, the program has implications for FVCs because during the time spent by professionals (the most relevant cost according to the cost analysis carried out), they would have carried out monitoring or accompanying activities not aimed at reducing the psychological impact of divorce. Therefore, there is an improvement in the utilization of the available resources. This has also meant greater recognition and added value for the centers and their professionals.

Fourth, variables such as parents’ age, gender or the support of their intervention group do not appear to be related to the results obtained during the study. Therefore, groups can be mixed in gender and age. Participants, indeed, were very satisfied with the process and the professionals of the FVCs applied the protocol without difficulties. FVCs will continue offering it as an intervention tool. This is important in order to scale up the program to new organizations.

As mentioned in the introduction, mental health and well-being are part of one of the SDGs established by the United Nations. This objective is even more important in the current era, because due to COVID-19, the number of people affected by symptoms of depression or anxiety is growing and, in people who already had symptoms, these are worsening [32]. In addition, the current crisis is having an impact on family routines and relationships and, due to these changes, divorces are expected to increase [10]. This increase, along with the potential danger of mental health problems at this time, makes programs like Egokitzen more necessary and makes the FVCs’ role throughout the process more relevant.

## 5. Conclusions

The Egokitzen program, apart from achieving good results in the community context showing a reduction in parental symptomatology and, indirectly, of child symptomatology, is economically efficient. The program achieves a return of EUR 3.10 for each euro invested in it.

## Figures and Tables

**Table 1:** Descriptive statistics by treatment group.

Variables	Comparison Group	Intervention Group	Total
No. of participants	112	270	382
Proportion of mothers (%)	57	60	61
Participants with joint physical custody (%)	23	21	22
No. of children			
Mean	1.47	1.56	1.54
SD	0.71	0.79	0.77
Age of parents			
Mean	40.90	41.28	41.18
SD	6.69	6.43	6.49
Min	26	23	23
Max	53	63	63
Age of children			
Mean	8.28	8.44	8.40
SD	4.11	4.37	4.30
Min	2	1	1
Max	19	20	20

**Table 2:** Cost valuation: resources, resource usage, and monetizing measure.

Resources	Resource Usage	Monetizing Measure
TRAINING		
TRAINERS	Average hours to train each group of FVC professionals	Trainer salary/hour
	Number of trainers in each group	
Participants (FVC professionals)	Average number of participants per training group	FVC Professional salary/hour
	Average hours in the training sessions	
Trips to perform the training	Trainers’ number of trips per training group	Cost of trip/group
Materials	Amount of materials delivered to each participant	Cost of materials/participant
INTERVENTION		
FVC Professionals	Number of FVC professionals in each parent group	FVC Professional salary/hour
	Hours of each FVC professional in each parent group	
Materials	Amount of materials delivered to parents	Cost of materials/participant
Administration (program management)	Administration staff hours for program management (hours/parent group)	AS salary/hour

**Table 3:** Average cost per group and total cost of the training and intervention phases.

Resources	Average Cost per Group of FVC Professionals (€)	Total Cost (€)
Training		
TRAINERS	237.97	1421.81
Participants (FVC professionals)	1299	7794
Trips to perform the training	294	1764
Materials	20	120
TOTAL TRAINING	1855.97	11,099.81
Resources	Average cost per group of parents (€)	Total costs (€)
Intervention		
FVC Professionals	1143.12	44,581.68
Materials	3.5	136.5
Administration(program management)	36.37	1418.43
TOTAL INTERVENTION	1182.99	46,136.61
TOTAL TRAINING + INTERVENTION		57,236.42

Source: Internal data of the project team and the FVC.

**Table 4:** Improvement or worsening in depressive symptomatology (in %).

Intervention Group				Control Group			
		POST				POST	
		*p* < T: No	*p* > T: Yes			*p* < T: No	*p* > T: Yes
PRE	*p* > T: Yes	17.30%	21.08%	PRE	*p* > T: Yes	5.56%	13.89%
	*p* < T: No	57.84%	3.78%		*p* < T: No	79.17%	1.39%

**Table 5:** Improvement or worsening in children’s anxiety symptomatology (in %).

Group	Improved	Worsened
Intervention	58.06%	25.81%
Control	50.00%	50.00%
NET EFFECT	8.06%	−24.19%

**Table 6:** Cost-benefit analysis of the intervention program.

Activity	No. of Participants	Costs	Benefit	Net Result	Benefit/€ Spent
Intervention	For every 100 participants	16,900 €	52,410 €	35,510	3.10
	For the entire sample of the study	46,136 €	143,081 €	96,945 €	
Intervention + Training	For the entire sample of the study	57,236 €	143,081 €	96,945 €	2.50

**Table 7:** Intervention sensitivity analysis.

Type of Change	No. of Participants Considered	Costs	Benefit	Net Income	Benefit/€ Spent
Change in costs	For every 100 participants	38,300 €	63,568 €	25,268 €	1.66
	For the entire sample of the study	104,515 €	173,540 €	69,025 €	
Change in symptomatology duration	For every 100 participants	16,900 €	26,206 €	9306 €	1.55
	For the entire sample of the study	46,136 €	71,542 €	25,406 €	

**Table 8:** Summary of results in three different scenarios (for every 100 participants).

Scenario	Assumptions		Costs	Benefit	Benefit/€ Spent
1	Costs	Real costs	16,900 €	52,410 €	3.10
	Benefits	Depressive symptomatology during 2 years (Layard et al. [29]) and anxiety in children during 6 months (Hathaway et al. [30] and Ministry of Health and consumption [31])			
2	Costs	Increase of human capital costs	38,300 €	63,568 €	1.66
	Benefits	Depressive symptomatology during 2 years [29] and anxiety in children during 6 months [30,31]			
3	Costs	Real costs	16,900 €	26,206 €	1.55
	Benefits	Conservative in benefits: depressive symptomatology during 1 year and anxiety in children during 3 months			

## Data Availability

The dataset associated with this submission is available at Mendeley Database https://doi.org/10.17632/cjj6d7ks7r.1 (accessed on 11 March 2022).

## References

[1] World Health Organization (2015). The European Mental Health Action Plan 2013–2020.

[2] United for Global Mental Health (2020). The Impact of COVID-19 on Global Mental Health: A Brief. https://unitedgmh.org/sites/default/files/2020-09/The%2BImpact%2BOf%2BCovid-19%2BOn%2BGlobal%2BMental%2BHealth%2BReport.pdf.

[3] United Nations (2020). Policy Brief: COVID-19 and the Need for Action on Mental Health. https://www.un.org/sites/un2.un.org/files/un_policy_briefcovid_and_mental_health_final.pdf.

[4] Braver S.L., Shapiro J.R., Goodman M.R., Fine M.A., Harvey J.H. (2006). Consequences of divorce for parents. Handbook of Divorce and Relationship Dissolution.

[5] Emery R., Tornello S. (2014). Rejoinder to: Who assumes the burden of proof when there is no neutral null hypothesis?. J. Marriage Fam..

[6] Garbarino S., Lanteri P., Durando P., Magnavita N., Sannita W.G. (2016). Co-Morbidity, Mortality, Quality of Life and the Healthcare/Welfare/Social Costs of Disordered Sleep: A Rapid Review. Int. J. Environ. Res. Public Health.

[7] Mahrer N., O’Hara K., Sandler I., Wolchik S. (2018). Does shared parenting help or hurt children in high-conflict divorced families?. J. Divorce Remarriage.

[8] (2020). Instituto Nacional de Estadística: Estadística de Nulidades, Separaciones y Divorcios. https://www.ine.es/prensa/ensd_2019.pdf.

[9] (2018). Instituto de Política Familiar: Informe Evolución de la Familia en Europa 2018. http://www.ipfe.org/Espa%C3%B1a/Documentos/IPF.

[10] Pietromonaco P.R., Overall N.C. (2020). Applying relationship science to evaluate how the COVID-19 pandemic may impact couples’ relationships. Am. Psychol..

[11] Uphold-Carrier H., Utz R. (2012). Parental divorce among young and adult children: A long-term quantitative analysis of mental health and family solidarity. J. Divorce Remarriage.

[12] Auersperg F., Vlasak T., Ponocny I., Barth A. (2019). Long-term effects of parental divorce on mental health—A meta-analysis. J. Psychiatr. Res..

[13] Van Dijk R., Van der Valk I., Deković M., Branje S. (2020). A meta-analysis on interparental conflict, parenting, and child adjustment in divorced families: Examining mediation using meta-analytic structural equation models. Clin. Psychol. Rev..

[14] Arbuthnot J., Gordon D.A. (1994). Does mandatory divorce education work: A six-month outcome evaluation?. Fam. Counc. Court. Rev..

[15] Sandler I., Gunn H., Mazza G., Tein J., Wolchik S., Berkel C., Jones S., Porter M. (2018). Effects of a program to promote high-quality parenting by divorced and separated fathers. Prev. Sci..

[16] Martínez-Pampliega A., Aguado V., Corral S., Cormenzana S., Merino L., Iriarte L. (2015). Protecting children after a divorce: Efficacy of Egokitzen—An intervention program for parents on children’s adjustment. J. Child Fam. Stud..

[17] Martinez-Pampliega A., Herrero M., Cormenzana S., Corral S., Sanz M., Merino L., Iriarte L., Ochoa de Alda I., Alcaniz L., Alvarez I. (2021). Custody and Child Symptomatology in High Conflict Divorce: Analysis of Latent Profiles. Psicothema.

[18] Malet-Larrea A., Goyenechea E., Gastelurrutia M.A., Calvo B., García-Cárdenas V., Cabases J.M., Noain A., Martínez-Martínez F., Sabater-Hernández D., Benrimoj S.I. (2017). Cost analysis and cost-benefit analysis of a medication review with follow-up service in aged polypharmacy patients. Eur. J. Health Econ..

[19] Foster E.M., Porter M.M., Ayers T.S., Kaplan D.L., Sandler I. (2007). Estimating the costs of preventive interventions. Eval. Rev..

[20] Derogatis L.R. (1992). SCL-90-R: Administration, Scoring and Procedures Manual for the Revised Version and Other Instruments of the Psychopathology Rating Scale Series.

[21] González de Rivera J.L., De las Cuevas C., Rodríguez M., Rodrıíguez F. (2002). Cuestionario de 90 síntomas SCL-90-R de Derogatis, Adaptación española [The Symptom Checklist 90 SCL-90-R. Spanish adaptation].

[22] Achenbach T.M. (1991). Manual for the Child Behavior Checklist and Profile.

[23] Chisholm D., Diehr P., Knapp M., Patrick D., Treglia M., Simon G. (2003). Depression status, medical comorbidity and resource costs: Evidence from an international study of major depression in primary care (LIDO). Br. J. Psychiatry.

[24] Sobocki P., Jönsson B., Angst J., Rehnberg C. (2006). Cost of depression in Europe. J. Ment. Health Policy Econ..

[25] Salvador-Carulla L., Bendeck M., Fernandez A., Albertí-Casas C. (2011). Costs of depression in Catalonia (Spain). J. Affect. Disord..

[26] Gustavsson A., Svensson M., Jacobi F., Allgulander C., Alonso J., Beghi E., Dodel R., Ekman M., Faravelli C., Fratiglioni L. (2011). Cost of disorders of the brain in Europe 2010. Eur. Neuropsychopharmacol..

[27] Pella J.E., Slade E.P., Pikulski P.J., Ginsburg G.S. (2020). Pediatric Anxiety Disorders: A cost of illness analysis. J. Abnorm. Child Psychol..

[28] (2020). Instituto Nacional de Estadística. https://www.ine.es/varipc/index.do.

[29] Layard R., Clark D., Knapp M., Mayraz G. (2007). Cost-benefit analysis of psychological therapy. Natl. Inst. Econ. Rev..

[30] Hathaway E.E., Walkup J.T., Strawn J.R. (2018). Antidepressant treatment duration in pediatric depressive and anxiety disorders: How long is long enough?. Curr. Probl. Pediatr. Adolesc. Health Care.

[31] (2006). Ministerio de Sanidad y Consumo: Guía de Práctica Clínica Para el Manejo de Pacientes con Trastornos de Ansiedad en Atención Primaria. https://portal.guiasalud.es/wp-content/uploads/2018/12/GPC_430_Ansiedad_Lain_Entr_compl.pdf.

[32] Salari N., Hosseinian-Far A., Jalali R., Vaisi-Raygani A., Rasoulpoor S., Mohammadi M., Rasoulpoor S., Khaledi-Paveh B. (2020). Prevalence of stress, anxiety, depression among the general population during the COVID-19 pandemic: A systematic review and meta-analysis. Glob. Health.

